# Deep learning-based left ventricular segmentation demonstrates improved performance on respiratory motion-resolved whole-heart reconstructions

**DOI:** 10.3389/fradi.2023.1144004

**Published:** 2023-06-02

**Authors:** Yitong Yang, Zahraw Shah, Athira J. Jacob, Jackson Hair, Teodora Chitiboi, Tiziano Passerini, Jerome Yerly, Lorenzo Di Sopra, Davide Piccini, Zahra Hosseini, Puneet Sharma, Anurag Sahu, Matthias Stuber, John N. Oshinski

**Affiliations:** ^1^ Wallace H. Coulter Department of Biomedical Engineering, Emory University and the Georgia Institute of Technology, Atlanta, GA, United States; ^2^ Digital Technology and Innovation, Siemens Medical Solutions USA, Princeton, NJ, United States; ^3^ Diagnostic and Interventional Radiology, Lausanne University Hospital, Lausanne, Switzerland; ^4^ Advanced Clinical Imaging Technology, Siemens Healthcare AG, Lausanne, Switzerland; ^5^ MR R&D Collaboration, Siemens Medical Solutions USA, Atlanta, GA, United States; ^6^ Department of Medicine, Division of Cardiology, Emory University School of Medicine, Atlanta, GA, United States; ^7^ Department of Radiology & Imaging Science, Emory University School of Medicine, Atlanta, GA, United States

**Keywords:** free-breathing, whole-heart CMR, motion compensation, deep learning 3D segmentation, LV volume

## Abstract

**Introduction:**

Deep learning (DL)-based segmentation has gained popularity for routine cardiac magnetic resonance (CMR) image analysis and in particular, delineation of left ventricular (LV) borders for LV volume determination. Free-breathing, self-navigated, whole-heart CMR exams provide high-resolution, isotropic coverage of the heart for assessment of cardiac anatomy including LV volume. The combination of whole-heart free-breathing CMR and DL-based LV segmentation has the potential to streamline the acquisition and analysis of clinical CMR exams. The purpose of this study was to compare the performance of a DL-based automatic LV segmentation network trained primarily on computed tomography (CT) images in two whole-heart CMR reconstruction methods: (1) an in-line respiratory motion-corrected (Mcorr) reconstruction and (2) an off-line, compressed sensing-based, multi-volume respiratory motion-resolved (Mres) reconstruction. Given that Mres images were shown to have greater image quality in previous studies than Mcorr images, we *hypothesized* that the LV volumes segmented from Mres images are closer to the manual expert-traced left ventricular endocardial border than the Mcorr images.

**Method:**

This retrospective study used 15 patients who underwent clinically indicated 1.5 T CMR exams with a prototype ECG-gated 3D radial phyllotaxis balanced steady state free precession (bSSFP) sequence. For each reconstruction method, the absolute volume difference (AVD) of the automatically and manually segmented LV volumes was used as the primary quantity to investigate whether 3D DL-based LV segmentation generalized better on Mcorr or Mres 3D whole-heart images. Additionally, we assessed the 3D Dice similarity coefficient between the manual and automatic LV masks of each reconstructed 3D whole-heart image and the sharpness of the LV myocardium-blood pool interface. A two-tail paired Student’s *t*-test (alpha = 0.05) was used to test the significance in this study.

**Results & Discussion:**

The AVD in the respiratory Mres reconstruction was lower than the AVD in the respiratory Mcorr reconstruction: 7.73 ± 6.54 ml vs. 20.0 ± 22.4 ml, respectively (*n* = 15, *p*-value = 0.03). The 3D Dice coefficient between the DL-segmented masks and the manually segmented masks was higher for Mres images than for Mcorr images: 0.90 ± 0.02 vs. 0.87 ± 0.03 respectively, with a *p*-value = 0.02. Sharpness on Mres images was higher than on Mcorr images: 0.15 ± 0.05 vs. 0.12 ± 0.04, respectively, with a *p*-value of 0.014 (*n* = 15).

**Conclusion:**

We conclude that the DL-based 3D automatic LV segmentation network trained on CT images and fine-tuned on MR images generalized better on Mres images than on Mcorr images for quantifying LV volumes.

## Introduction

The left ventricle (LV) acts as a pump to send oxygenated blood from the heart to the rest of the body. Many cardiac pathologies including heart failure, valvular disease, and congenital abnormalities involve LV remodeling that changes the shape and function of the LV (1–3). 3D segmentation of LV volume has an important role as a clinical metric as well as in 3D printing to make image phantoms (4) and computational fluid dynamics simulations (5). Cardiac magnetic resonance (CMR) is the current standard for assessing ventricular volume in both adults and pediatric patients; CMR provides more accurate and reproducible ventricular volume quantifications when echocardiography images are ambiguous (2, 6). Accurate volume measurement with CMR requires precise contouring of the endocardial borders. Due to the variability of the heart shape and myocardial motion patterns across patients and within the same patient at different time points, ventricular segmentation is a nontrivial task (6).

Currently, expert manual segmentation is often used to delineate the endocardial borders, usually based on a set of short-axis slices (7, 8). However, manual segmentation is cumbersome, time-consuming, and subject to inter- and intra-observer variability (9). There are continuous efforts to develop automated processes to meet the clinical need for faster and more accurate segmentation. In addition to the model-based semi-automatic segmentation methods (10–14), machine learning (ML)- and deep learning (DL)-based methods have been developed that utilize large labeled data sets to train a deep neural network for automatic segmentation of 2D or 3D image sets (6, 8). Segmentation accuracy can be greatly reduced in the presence of motion that degrades image quality, causes artifacts, or results in blurring of the blood-myocardial boundaries (8, 15, 16). Thus, there is a need to reduce motion artifacts in underlying images in order to improve the performance of ML- or DL-based LV segmentation.

Current CMR methods to compensate for respiratory motion include breath-holding for 2D imaging or highly accelerated 3D imaging, and free-breathing approaches for 3D whole-heart imaging using navigators for respiratory gating or respiratory motion correction (17, 18, 19). More recently, respiratory motion has been extracted from the acquired data and used to resolve the acquired k-space data into different respiratory motion-resolved bins and perform reconstruction using compressed sensing (CS) [i.e., XD-GRASP (20)]. To our knowledge, there are no studies evaluating the performance of DL-based networks on images from these different reconstruction methods used to compensate for motion artifacts. Thus, in this study, we evaluated the performance of a previously validated DL-based automatic segmentation to assess the accuracy of LV segmentation for two kinds of respiratory motion-compensated images: (1) in-line, motion-corrected (Mcorr) (19), and (2) off-line, compressed sensing-based, multi-volume motion-resolved (Mres) (20). The 3D DL-based automatic segmentation network used in this study was not trained on Mcorr or Mres images but primarily on computed tomography (CT) images. Therefore, another goal in this study was to investigate the generalizability of a network trained primarily on cardiac CT images to be applied on motion-corrected and motion-resolved MR images.

The difference between the automatically segmented LV volumes and manually segmented LV, or the absolute volume difference (AVD), is the primary metric for accuracy assessment. We also calculated the 3D Dice coefficients and edge sharpness for the two LV reconstruction methods as more traditional metrics to assess and interpret segmentation accuracy. It was shown in previous studies (20, 21) that Mres images have greater subjective image quality than Mcorr images, meaning the endocardial borders could be more accurately delineated in Mres vs. Mcorr. Therefore, we hypothesized that the use of an off-line, compressed sensing-based, Mres XD-GRASP reconstruction method would provide more accurate DL-based LV segmentation than an in-line Mcorr method, as assessed by the AVD between the DL-based model and expert manual segmentation.

## Methods

### Image acquisition

**Figure 1:**
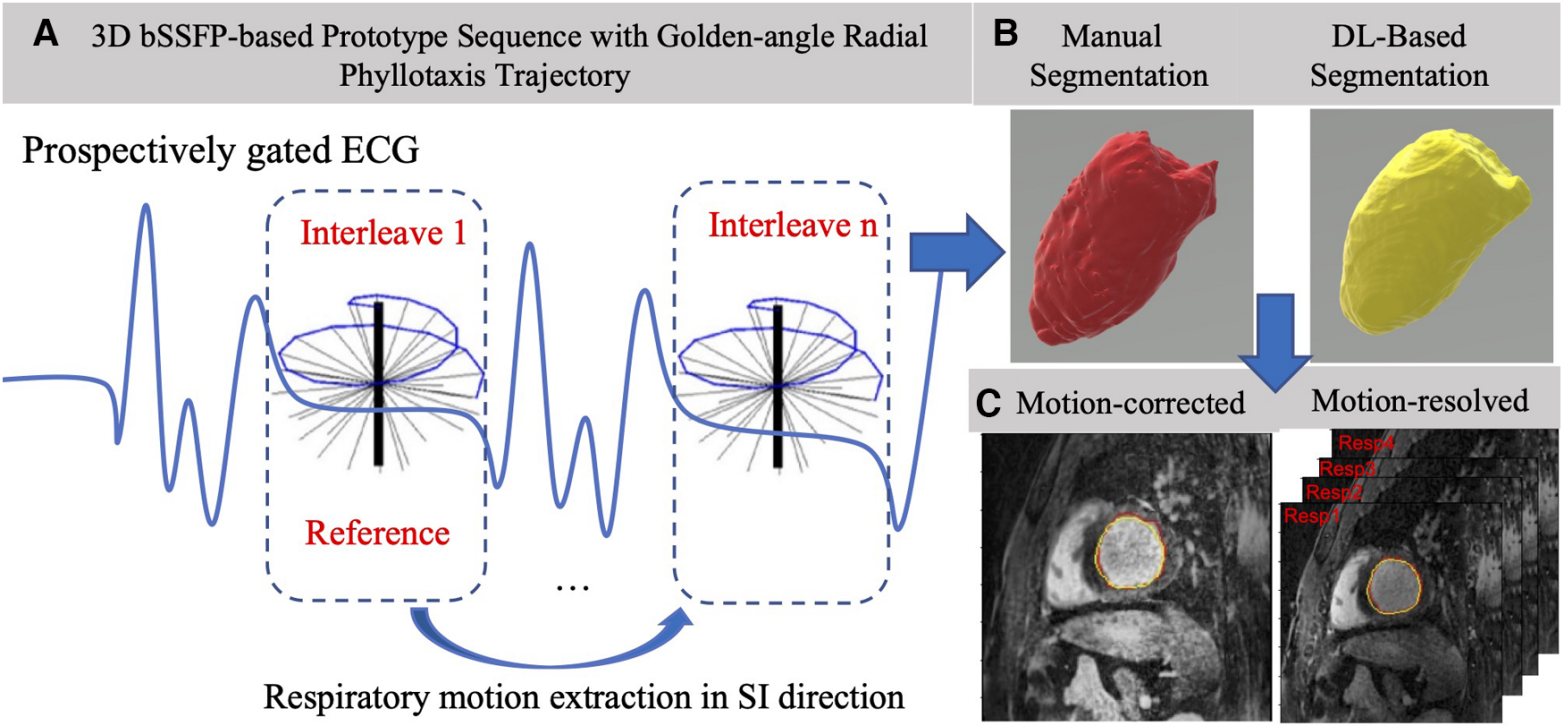
Overview of the acquisition, segmentation, and analysis pipeline. (**A**) Left: 3D motion-corrected data is acquired using a bSSFP sequence using a golden-angle radial phyllotaxis trajectory in mid-diastole gated by a synchronously acquired ECG signal. The motion-corrected image is reconstructed in-line on the scanner after acquisition and the raw data are exported for motion-resolved reconstruction resulting in four whole-heart volumes, each of which corresponds to a distinctive respiratory phase. (**B**) Top right: After reconstruction, five volumes are exported to the I21-CVAE 3D whole-heart segmentation network for automatic segmentation of all cardiac chambers, and the manual segmentation is done on Mimics. (**C**) Bottom right: Automatic (yellow) and manual (red) segmented masks overlaid on one motion-corrected and four motion-resolved images per patient to compute the Dice coefficient and quantify LV volumes.

The study was performed on 15 patients undergoing a clinically indicated CMR scan at Emory University Hospital on a 1.5 T scanner (MAGNETOM Avanto^FIT^, Siemens Healthcare, Erlangen, Germany). The study cohort included seven adult patients with congenital abnormalities, four patients with heart failure, and four patients with valvular disease. The study was approved by the Institutional Review Board of Emory University and patients provided informed consent. Images were acquired using an 18-channel body array coil in combination with the spine array coil and a saturation slab at the level of the anterior chest wall. Data were acquired with a prototype ECG-gated 3D golden-angle radial phyllotaxis interleaved balanced steady state free precession (bSSFP) sequence with fat saturation, T2 preparation, and 10 ramp-up pulses to achieve steady states over 220 mm × 220 mm × 220 mm field of view with matrix size of 192 × 192 × 192 per readout, giving an isotropic spatial resolution of 1.14 mm × 1.14 mm × 1.14 mm (Figure 1A). Excitation used a 115° flip angle, and a total of 12,224 radial lines were acquired with 32 segments per interleaf. Acquisition of each interleaf occurred over 200 ms with a TE of 1.55 ms and a bandwidth of 1000 Hz/pixel.

### Motion compensation and image reconstruction

**Figure 2:**
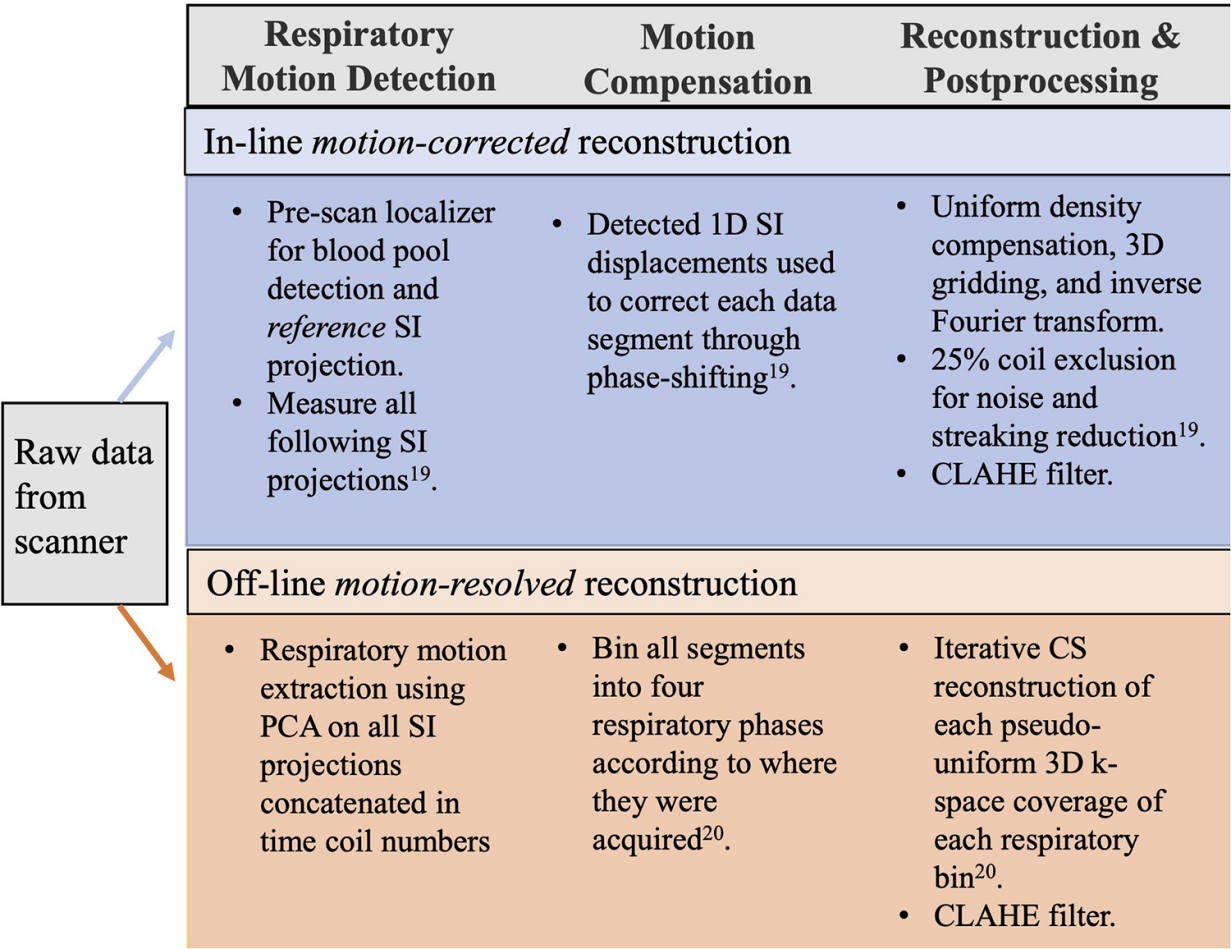
Pipeline for in-line motion-corrected and off-line motion-resolved reconstructions. Motion-corrected images were reconstructed in-line on the scanner. After intensity normalization, the bright signal from the heart was isolated and segmented from the other bright signals from the anterior chest wall, spine, and lateral structures in the reference SI projection and cross-correlated with the following SI acquisitions for 1D respiratory motion compensation using phase shifting (19). The reconstruction of motion-corrected images was fully integrated into the Siemens data acquisition and image reconstruction framework. After reconstruction, a 3D CLAHE filter was added. The raw data were exported off-line for the motion-resolved reconstruction using a customized MATLAB program. The algorithm for motion-resolved reconstruction first identified the respiratory motion signal using principal component analysis as a time-varying signal that lies within typical respiratory frequency ranges from a matrix of concatenated SI k-space projections of all coils. After motion extraction, k-space data were binned into four respiratory phases and CS was used to reconstruct each of the four highly under-sampled respiratory phase image sets. In this study, the regularization parameter λ was 0.1; this value was selected to balance the data consistency and smoothness in the final reconstructed image. The reconstructions were performed using a server equipped with two 24-core CPUs, 384 GB RAM, and an 11 GB NVIDIA GPU. After reconstruction, a 3D CLAHE filter was added. SI, superior-to-inferior; CLAHE, contrast limited adaptive histogram equalization; CS, compressed sensing.

The reconstruction pipeline for the Mcorr and Mres images is shown in Figure 2. Mcorr images are 1D respiratory motion corrected in the superior-to-inferior (SI) direction and reconstructed in-line on the scanner, as previously described (19). Briefly, the repeatedly acquired SI projections are each cross-correlated with the reference SI projection to find the phase shift needed to correct for respiratory motion in the free-breathing acquisition. The raw data were exported off-line for respiratory motion extraction and binning into four respiratory bins for under-sampled reconstruction of the Mres images (20, 22) on MATLAB (R2020b, MathWorks Inc., Matick, MA, United States). After reconstruction, a single static phase 3D whole-heart image was reconstructed per patient using Mcorr image reconstruction and four distinct 3D whole-heart images were reconstructed, each from a different respiratory phase bin using Mres reconstruction. Contrast limited adaptive histogram equalization (CLAHE) filtering was added after reconstruction for both Mcorr and Mres images to ensure their contrast variation would be treated equally in both image sets.

### LV segmentation

**Figure 3:**
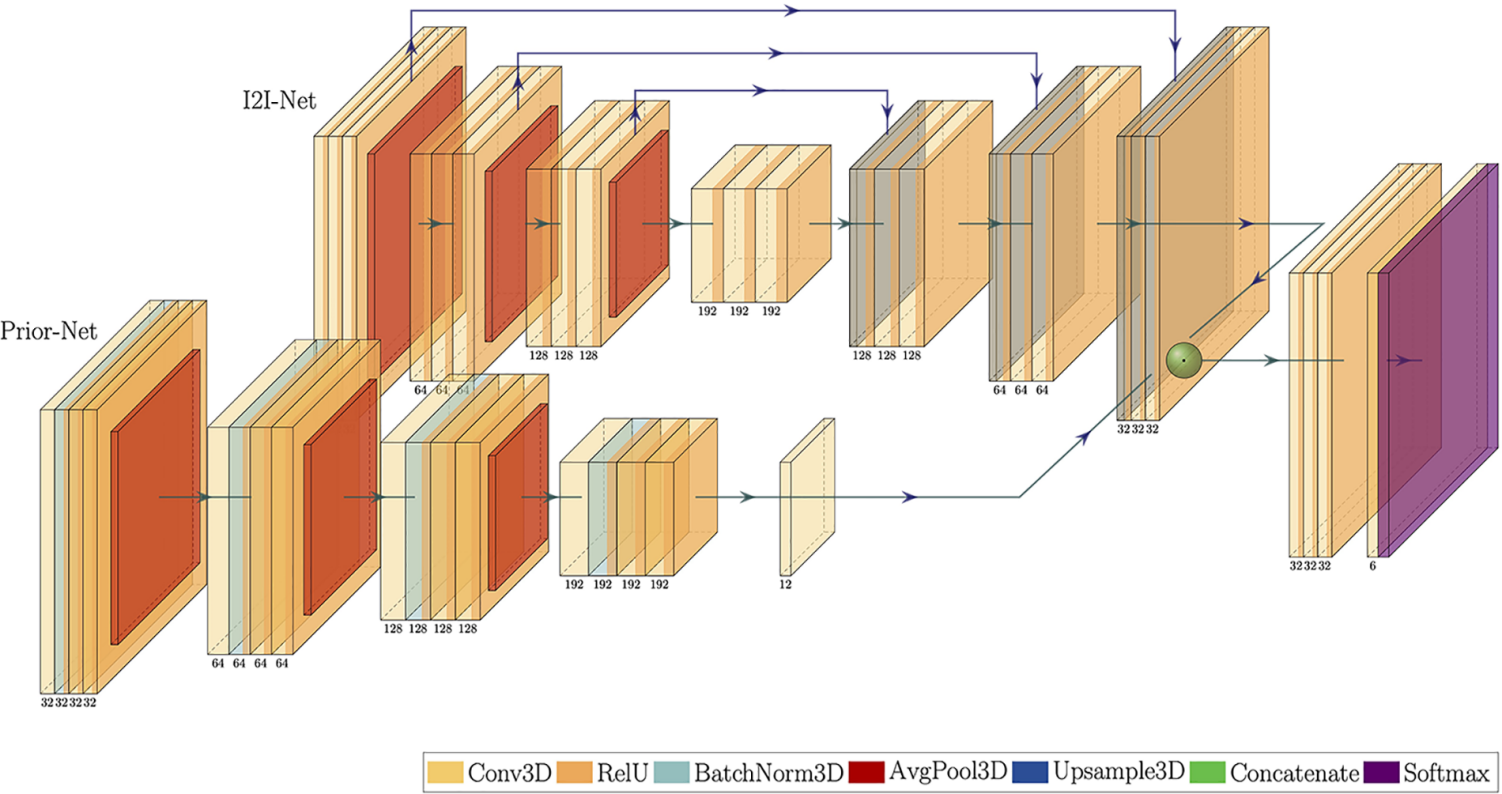
Transfer learning I2I-CVAE neural network architecture. The Prior-Net is an encoder that learns the latent space of the images and was trained using CT images for strong shape priors of the cardiac chambers. The I2I-Net is an encoder–decoder network for 3D whole-heart segmentation.

The respiratory Mcorr and four Mres whole-heart image volumes were segmented using a 3D image-to-image DL network combined with a conditional variational autoencoder (I2I-cVAE) (23), trained through transfer learning (24). The network architecture is shown in Figure 3. The I2I network and cVAE networks were trained simultaneously with a combined cross entropy loss (standard segmentation loss) and Kullback–Leibler divergence loss. Such a training approach enables the network to not only learn a mapping from image to segmentation, but also to learn a latent space, encoding the joint distributions of images and possible segmentations. A latent space dimension of six was observed to provide optimal results. During inference, the algorithm uses only the encoder block of the cVAE network, also referred to as the prior network, and samples the mean of the distribution, which is combined with features from the I2I network to create the final segmentation. The network was first trained on a large collection of 1,059 cardiac gated calcium scoring CT datasets (25) and annotated segmentation masks for the cardiac chambers to learn strong shape priors of the chambers. The pretrained weights were then fine-tuned using multi-vendor MR data consisting of 122 patient volumes, covering a variety of 3D MRI sequences (26). The training 3D MR datasets did not contain Mcorr or Mres images. In the current study, the LV was segmented manually on the Mcorr image and four Mres images for each patient by a trained expert using Mimics (Materialize, Inc.) (Figure 1B).

### LV volume comparison and Dice similarity coefficients

The AVD between the manually and DL-segmented LV was computed for all five image sets per patient (one for the in-line Mcorr reconstruction and four different respiratory bins for the off-line Mres reconstruction). The minimum AVD from the four respiratory phases in the off-line Mres reconstruction was compared with the AVD for the in-line Mcorr method (Figure 1C) using a paired *t*-test with alpha = 0.05. The 3D dice similarity coefficient (DSC) (27) was computed using the seg-metrics (28) package in Python between the manual and DL-segmented LV borders for all five image sets. The maximum 3D DSC of the four respiratory phases for the off-line Mres segmentations was compared with the DSC of the in-line Mcorr segmentations using a paired *t*-test with alpha = 0.05.

### LV-blood pool sharpness measurements

To interpret the potential difference in the LV segmentation performance of the 3D DL-based network, an image quality-based metric, the LV endocardial border sharpness, was included. The sharpness measurement was used to assess the potential connection between the superior performance with image quality of the Mcorr and Mres images. LV sharpness measurement was performed using a custom-written program in MATLAB based on a previously presented method (29). The details of the quantification process are illustrated in Supplementary Figure S1.

## Results

For each set of Mres images (3D + 4 respiratory states), the respiratory state with the lowest AVD or the greatest 3D DSC was chosen as the best static Mres image for comparison with the Mcorr images. The respiratory state with the greatest mid-SA slice sharpness was used as the representative static Mres image for comparison with the Mcorr image.

**Figure 4:**
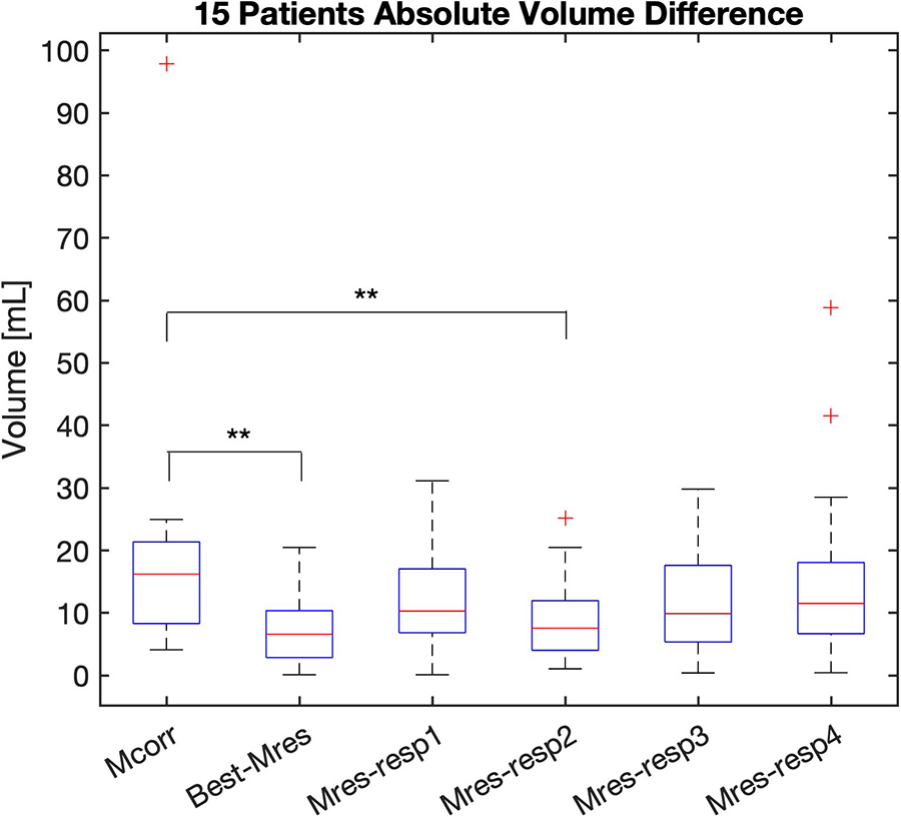
AVD of LV DL-based segmentation on the Mcorr image and each respiratory state Mres image. The AVD of Mcorr (Mcorr), Best-Mres, and four respiratory state Mres (Mres-resp1–Mres-resp4) images are shown in each boxplot. A two-tail paired Student’s *t*-test was used to test the significance of the difference between the two methods. Significance was found between Mcorr and Best-Mres images (*p* = 0.03) as well as Mcorr and second respiratory state (during inspiration) Mres images (*p* = 0.04). AVD, absolute volume difference; Mcorr, motion-corrected; Mres, motion-resolved; Best-Mres, best respiratory state Mres.

The LV segmentations of the best-Mres images (*n* = 15) had a lower AVD than that of the Mcorr images (*n* = 15), 7.7 ± 6.5 ml vs. 20.0 ± 22.4 ml, respectively (*p* = 0.03) (Figure 4). The AVDs of the LV segmentations on the Mcorr images vs. on each phase of the Mres images are also shown in Figure 4. The LV segmentation of respiratory phase 2 of the Mres images have a lower AVD than that of the motion-corrected images (*p* = 0.04) (Supplementary Table S1). This result indicates that Mres images enabled the DL network to more accurately find the correct LV volume than the Mcorr images. The lowest AVD respiratory state was found primarily on the second respiratory phase (during expiration) in 8/15 patients, on the first respiratory phase (during end expiration) in 5/15 patients, on the third respiratory phase (during inspiration) in 1/15 patients, and on the fourth respiratory phase (during inspiration) in 1/15 patients. Note that one patient (subject 1 in Supplementary Figure S2) had a larger difference in volume of the automatic vs. manual Mcorr segmentations. We tested if removing this subject would affect the results; even after removing the subject from the analysis, the results were still significant for the AVD between the Mcorr and Mres images over the remaining group.

**Figure 5:**
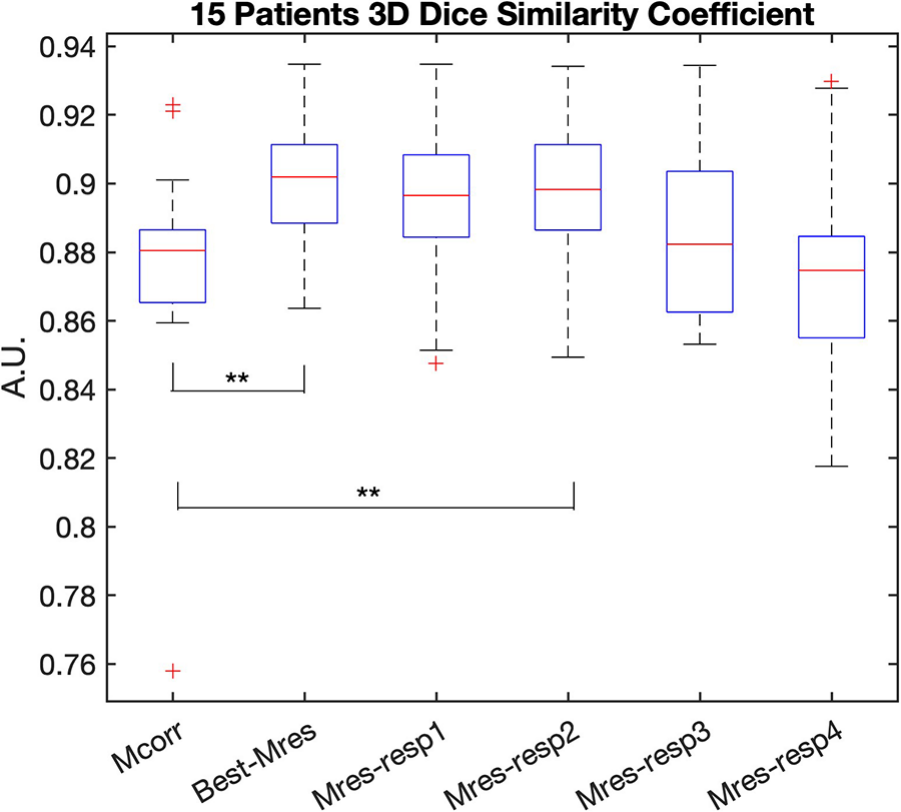
3D DSC of LV DL-based segmentation on the Mcorr image and each respiratory state Mres image. The 3D DSC of Mcorr (Mcorr), Best-Mres, and four respiratory state Mres (Mres-resp1–Mres-resp4) images are shown in each boxplot. A two-tail paired Student’s *t*-test was used to test the significance of the difference between the two methods. Significance was found between Mcorr and Best-Mres images (*p* = 0.02) as well as Mcorr and second respiratory state (during inspiration) Mres images (*p* = 0.049). DSC, dice similarity coefficient; Mcorr, motion-corrected; Mres, motion-resolved; Best-Mres, best respiratory state Mres.

The LV segmentation of the representative static Mres images (*n* = 15) had a greater 3D DSC than that of the Mcorr images (*n* = 15), 0.90 ± 0.02 vs. 0.87 ± 0.03, respectively (*p*-value = 0.02) (Figure 5). The 3D DSCs of LV segmentation of the Mcorr images vs. each phase of Mres images are also shown in Figure 5. The LV segmentations of respiratory phase 2 of the Mres images have greater 3D DSCs than those of the Mcorr images (*p* = 0.049) (Supplementary Table S1). This result shows that the DL-segmented masks spatially overlap better with the manually annotated masks for Mres images than for Mcorr images. The greatest 3D DSC respiratory state was found primarily on the second respiratory phase in 8/15 patients, on the first respiratory phase in 5/15 patients, on the third respiratory phase in 1/15 patients, and on the fourth respiratory phase in 1/15 patients.

**Figure 6:**
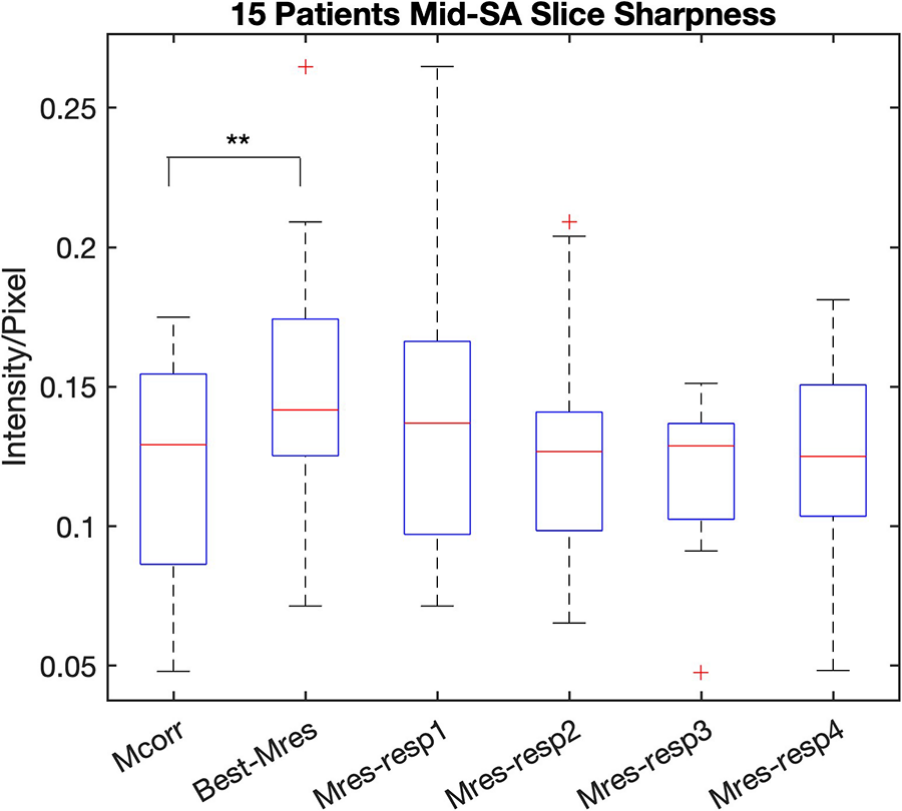
LV endocardial border sharpness of LV DL-based segmentation on the Mcorr image and each respiratory state Mres image. The LV endocardial border sharpness of Mcorr (Mcorr), Best-Mres, and four respiratory state Mres (Mres-resp1–Mres-resp4) images are shown in each boxplot. A two-tail paired Student’s *t*-test was used to test the significance of the difference between the two methods. Significance was found between the Mcorr and Best-Mres images (*p* = 0.014), but no significant difference was found between Mcorr images and any Mres images for a single respiratory state. DL, deep learning; Mcorr, motion-corrected; Mres, motion-resolved; Best-Mres, best respiratory state Mres.

The mid-SA slice sharpness was greater for the representative static Mres images (*n* = 15) than for the Mcorr images (*n* = 15), 0.15 ± 0.05 vs. 0.12 ± 0.04, respectively (*p*-value = 0.014) (Figure 6). The LV endocardial border sharpness results of the Mcorr images vs. each respiratory phase of the Mres images are also shown Figure 6. There was no statistical significance found between the sharpness of the Mres images and Mcorr images for each respiratory state (Supplementary Table S1). The greatest mid-SA slice sharpness respiratory state was found primarily on the first respiratory state in 7/15 patients, on the second respiratory phase in 4/15 patients, on the fourth respiratory state in 2/15 patients, and on the third respiratory state in 2/15 patients.

The AVD and the 3D DSCs of the Mcorr images with respect to the LV segmentations of each of the four Mres images and middle-short-axis (Mid-SA) slice sharpness of these images are summarized in Supplementary Table S1. Respiratory phase 2 of the Mres images has a lower AVD (*p* = 0.04) and greater 3D DSC than the Mcorr images (*p* = 0.049).

## Discussion

This study evaluated the generalizability of a 3D DL-based neural network trained primarily on cardiac CT images to segment the LV from (1) in-line Mcorr MR images, and (2) off-line compressed sensing reconstructed Mres MR images. The main finding of this study was that the 3D DL-based LV segmentation network identifies an LV volume that is closer to the reference standard volume when using Mres MR images than when using Mcorr MR images. Importantly, this was a network trained primarily on cardiac CT images and did not include either Mcorr or Mres images in training database.

Contrary to many previous studies, this study did not seek to develop a new DL network, but rather sought to use an established DL segmentation network to evaluate how motion-compensation methods in the MR images affect the accuracy of the segmentation. The result that Mres images came closer to the reference standard than Mcorr images implies that the DL network found more similar features in the Mres images than motion-corrected images compared to the cardiac CT training dataset. Since cardiac CT images have very high contrast and excellent resolution, the similar features in the Mres images and cardiac CT images could be interpreted as indicating that Mres images have a higher image quality than Mcorr images. This interpretation illustrates an example of multi-modality transfer learning. The results could also be interpreted as indicating that the performance of a generic DL segmentation network on unseen 3D whole-heart images could be used as a method to judge underlying image quality, although the image quality results would only be valid for this specific network. In either interpretation, improvements in the DL network through increased training and improvements in the underlying image acquisition go hand-in-hand for improving CMR. As DL-based automated segmentations methods gain in popularity, it will be increasingly important to find image acquisition and reconstruction methods that specifically increase DL accuracy or provide close enough initial segmentations for finding clinically relevant metrics such as LV volume.

The AVD and 3D DSC metrics evaluated the volumetric and spatial overlap between the DL-segmented and the manually annotated LV. We found that the AVD between the DL-based and manually segmented LV was lower in the representative Mres data than in the Mcorr images. In addition, the 3D DSC of DL-based automatic segmentation and manual segmentation was higher for the representative Mres images than for the Mcorr images, implying that not only was the volume in the motion-resolved images closer to manual expert segmentation, but the spatial overlap between the volumes was also closer.

We found that the respiratory phase with the smallest AVD and the greatest 3D DSC was primarily respiratory phase 2, which is during the end expiration phase. We also found that respiratory phase 2 of the Mres images has a lower AVD and higher 3D DSC than that of the Mcorr images (Supplementary Table S1). It has been reported that the second respiratory phase has the least amount of relative respiratory displacement among the four respiratory phase-resolved reconstructions (20). These findings suggest that the DL-based LV segmentation method is more likely to be closer to manually annotated segmentation when the image exhibits less residual motion. Our result on LV endocardial border sharpness also agrees with this finding that Mres images have greater sharpness than Mcorr images, though no significant difference was found in the sharpness of Mcorr images and Mres images for respiratory phase 2 . We think that other image quality factors might also affect the performance of the DL network in LV segmentation, such as image homogeneity, and that LV endocardial border sharpness might not be the sole reason the specific DL-based segmentation network delineates an LV endocardial border that is closer to the expert manual segmentation.

Other factors could also affect the performance of DL-based segmentation on images generated by the two reconstruction methods. The Mcorr image cases with the worst performance on this specific DL network could be attributed to various reasons related to the reconstruction algorithm of Mcorr, including (19) (1) the presence of bright structures on the top of the segmented heart might negatively affect the accuracy of cross-correlation in Mcorr; (2) bright signals from the anterior chest wall might be insufficiently suppressed in some patients; (3) the 1D rigid-body motion model might be insufficient in modeling motion, therefore leading to residual motion artifacts in the images. Contrary to the cross-correlation method of Mcorr, Mres reconstruction extracts the respiratory motion signal that has the greatest contribution to the variation of the SI projection and therefore could be less affected by static bright structures. In one subject, the DL-based segmentation failed on the Mcorr image. In this subject, the LV was highly dilated and exhibited non-typical anatomy. Using the Mres images, the segmentation performed better (Subject 1 in Supplementary Figure S2). We suspect that this is due to the smoothing effect of the total variation compressed-sensing reconstruction of Mres images, which makes the dark artifact in the center blood pool smooth and therefore easier to categorize as the LV in the motion-resolved image.

The results of this study agree with findings from Stroud et al. (21), where they compared magnetic resonance angiography (MRA) exams in the aorta using Mcorr and Mres reconstructions. In that study, more traditional image quality grading by a radiologist was the primary metric for evaluation. Mres images were found to be superior to respiratory-compensated images. A study by Piccini et al. (30) used a deep convolutional neural network (DCNN) to directly assess image quality in free-breathing-Mres whole-heart images. The study showed that the respiratory image with the best image quality (as identified by an expert reader) could be directly determined with the network. This type of network could be used in combination with the method used in our study to automatically determine the best respiratory motion phase and then segment the LV volume on that phase. The methodology presented here could be extended to dimensions higher than 4D. Recently, a 5D free-running implementation of motion-resolved 3D whole-heart imaging has been presented (31). The 5D method reconstructs the 3D whole-heart images in both the cardiac and respiratory dimensions so that LV function can be characterized by measuring ejection fraction. Extension of the DL-based LV segmentation methodology presented in this work could be used on the 5D free-running imaging to automatically determine LV ejection fraction with high level of accuracy.

Since LV volume is a commonly used clinical indicator for the assessment of progression of cardiac diseases (32, 33), the ability to segment LV volumes accurately and rapidly is critical to patient assessment (34). Therefore, a volume-based DL segmentation could become a new metric for comparing the image quality of CMR images from various reconstruction and/or acquisition methods. Conventional CMR image quality assessments include measures such as the root mean-squared error (RMSE), structural similarity index (SSIM), peak signal-to-noise ratio (PSIR), and image sharpness, or by using expert grading (30, 35). These metrics look at underlying image quality metrics but do not specially look at a clinical metrics such as accurate LV volume determination.

The DL-based segmentation network presented in the study could also be used to automatically segment the right ventricle, right atrium, and left atrium. In this work, we chose to study the agreement between the DL-based and manual LV segmentation because the LV has the most defined regions where segmentation should start and end within the structure (i.e., aortic and mitral valve annuli). Other chambers have more complex morphology and boundaries that are more difficult to determine (36). We also chose to focus on LV segmentation because the patient cohorts involved in this study mostly have cardiac myopathies that already exhibit or could potentially develop LV remodeling, including hypertrophic myopathy and congenital heart diseases (coarctation and tetralogy of Fallot). Therefore, clinically, we are mostly interested in looking at the change in the size of the LV in these patients.

This study has limitations. This study used a limited number of clinical patients with various cardiac pathologies including tetralogy of Fallot and a bicuspid aortic valve. The prototype ECG-gated 3D golden-angle radial phyllotaxis interleaved bSSFP sequence acquires the data in 6–8 min, which was difficult to add to clinically indicated CMR scans for cardiac patients. Although the experiment size is small, it was not intended to train the network, only to evaluate the underlying motion artifacts and image quality in the two methods. Because of the small cohort size (*n* = 15) and the varied etiologies, we could not conclude whether Mres images are superior to Mcorr images for DL-based segmentation of the LV for specific pathologies. The Mcorr and Mres images were not included in the training since the goal of this study was to test the generalizability of a LV segmentation network on new datasets. Importantly, the findings in this study are based on a *single* DL automatic segmentation network and it may not be generalized until it has been tested on other 3D DL-based segmentations.

## Conclusion

Using a 3D whole-heart DL-based algorithm trained primarily on CT images, LV volumes segmented automatically on Mres MR images are closer to expert manual segmentations than those on Mcorr MR images. This study illustrates that multi-modality transfer learning in ML can be used in CMR and may be used as a tool to evaluate image quality in the acquired images.

## Data Availability

The raw data supporting the conclusions of this article will be made available by the authors, without undue reservation.
