# Supplementary material for: Deep learning-based left ventricular segmentation demonstrates improved performance on respiratory motion-resolved whole-heart reconstructions
Source: Front Radiol. 2023 Jun 2;3:1144004. doi: 10.3389/fradi.2023.1144004 (PMC10365088; doi:10.3389/fradi.2023.1144004)
Supplement: Supplementary file 1 [file Datasheet1.pdf]

Supplementary Material

1 Supplementary Figures and Tables

1.1 Supplementary Table

	Motion-corrected	Best Motion-resolved Respiratory State	Motion-resolved Respiratory State 1	Motion-resolved Respiratory State 2	Motion-resolved Respiratory State 3	Motion-resolved Respiratory State 4
Absolute Volume Difference AVD (ml)	20.0 +/- 22.4	7.73 +/- 6.54 (p* = 0.03)	12.6+/- 8.7 (p = 0.21)	**9.72 +/- 7.09 (p* = 0.04)**	12.5 +/- 9.8 (p = 0.12)	16.1 +/- 15.7 (p = 0.42)
Dice Similarity Coefficient DSC	0.87 +/- 0.03	0.90 +/- 0.02 (p* = 0.02)	0.90 +/- 0.03 (p = 0.09)	**0.90 +/- 0.02 (p* = 0.049)**	0.89 +/- 0.03 (p = 0.25)	0.87+/- 0.03 (p = 0.91)
Mid-SA Slice Sharpness	0.12 +/- 0.04	0.15 +/- 0.05 (p* = 0.014)	**0.14 +/- 0.05 (p = 0.22)**	0.13 +/- 0.04 (p = 0.41)	0.12 +/- 0.03 (p = 0.79)	0.13 +/- 0.04 (p = 0.68)

Supplementary Table 1: Absolute volume difference, 3D Dice Similarity Coefficients, and myocardium-to-blood pool images sharpness on a mid-short axis slice are shown for each motion resolved respiratory phase. The reconstruction with the best performance for each metric is in bold text.

1.2 Supplementary Figure

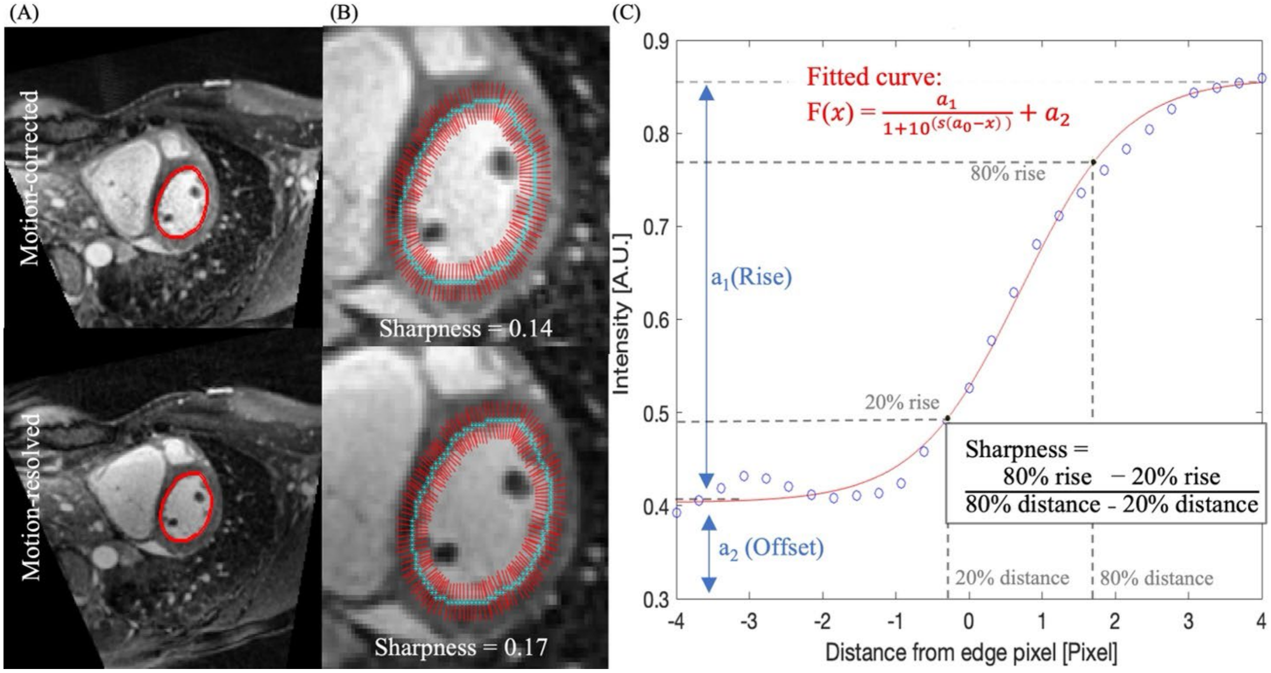

Supplementary Figure 1. Mid-SA slice LV sharpness Measurement. In each subject, the inline motion-corrected image volume segmentations and four offline motion-resolved image volume segmentations were reformatted to produce a mid-short-axis (mid-SA) oblique slice. (A) The motion-corrected and a single respiratory state motion-resolved image volumes are registered and reformatted into mid-SA slice and are overlaid with the endocardial mask (red) from the DL-based automatic segmentations. The AVD of the motion-corrected vs. motion-resolved is 16.4mL vs. 10.3mL, respectively, and the DCE of the Mcorr vs. Mres is 0.86 vs 0.89 respectively. (B) The automatically segmented mask images were used to locate LV endocardial contour (white) and the LV sharpness on each of the mid-SA slice was computed along the endocardial border at each edge pixels (cyan) in the direction of the edge gradient (red). (C) Example single edge pixel sharpness measurement. The image pixel values along the line segment plotted against the distance from the edge pixel (blue) and fitted to a non-linear function (red) f$(x) = \frac{a_1}{1+10^{(s(a_0-x))}} + a_2$ where a_1 denotes vertical rise range of the sigmoid and a_2 denotes the vertical offset. The image sharpness is computed as the change in intensity against the change in distance at the linear section of the fitted sigmoid curve, which is approximated as the section from 20% vertical rise to 80% vertical rise from the baseline.

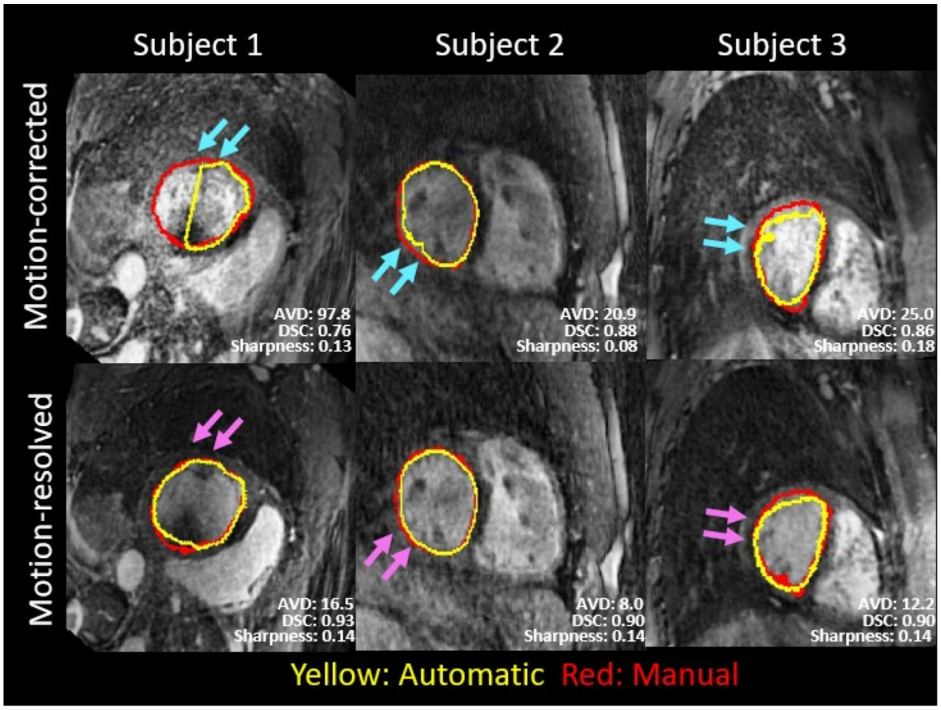

Supplementary Figure 2. Automatic and Manual Segmentation of Motion-corrected and Motion-resolved Images on three example subjects. The agreements between the automatic and manual segmented masks are shown for the automatic (yellow) and manual (red) segmented masks of three example patients with motion corrected images (top row) motion-resolved images with the least absolute volume difference (bottom row). The cyan arrows are point at the mis-alignments of automatic and manual segmented masks on motion corrected images while the magenta arrows on motion-resolved images.
